# Annotations

**Published:** 1902-12-06

**Authors:** 


### Annotations.

### Aliens, Education and Trachoma.

Years ago, when the prevalence of trachoma in various metropolitan poor-law schools led to a great outcry, many people were inclined to hold that the disease, as it existed in London at any rate, was a special product of "institutionalism," a special curse upon "barrack schools." A fuller knowledge, however, showed that although there was much in certain of the domestic arrangements of these schools by which the spread of the disease was facilitated, it was the constant influx of diseased children from outside which rendered it so difficult to eradicate the disease, and now, with some years of experience of compulsory elementary education, it is becoming clear that in addition to the problems connected with the more widely recognised infections, such as those of ringworm, scarlet fever, and other zymotic diseases, the question of trachoma will have to be faced by the managers of elementary as well as of poor-law schools. And the problem is no slight one. The more strictly we apply the broom, the more carefully we sweep into our elementary schools the waifs and strays, to deal with whom was one of the original objects of the Education Acts, the more forcibly are we impressed with the injustice of compelling decent, cleanly, uninfected children to sit side by side with others who are charged with the germs of many diseases. Nor can we fail to see that the problem is still further complicated by the unrestricted influx of aliens. It is only when a nation decides to keep out "undesirables" that it discovers how large a proportion of aliens fall into this category. America has found this out, Canada is beginning to discover it. According to the New York *Medical Record* the increase of trachoma in New York is causing much anxiety, and is attributed in part at least to the large number of immigrants who have been allowed to land while suffering from this disease. Since the beginning of the school year about 14,000 pupils have been excluded from school, 6,667 of whom were suffering from eye trouble. It is held that the prevalence of infective trachoma amongst the Austrian and Russian immigrants is largely responsible, and a demand is being made for the exclusion of all who are affected. If these trachoma cases are refused admission to the States we know pretty well where they will seek asylum, much to the complication of our educational problems.

### Infantile Mortality in Liverpool.

The fact that every community consists of a mixture of all sorts and conditions of men tends to smooth away many of the harsh lessons which a study of public health statistics might otherwise inculcate. In his recently published annual report Dr. E. W. Hope, medical officer for Liverpool, while giving the death-rate of that city as 21·6 per 1,000, shows that although in one part, namely, the Sefton Park division, it was only 11·8 per 1,000 in another, the Scotland division, it was no less than 32 per 1,000. But the lessons taught by death-rates, even when these are estimated for quite small areas, are apt to fall short of the truth. One wants, if possible, to group together for statistical purposes families which seem, if one may so say, to affect certain causes of death. This Dr. Hope has done in a way which, although doubtless open to criticism, gives results which are horribly striking. Taking consecutively every family in which the death of an infant occurred, he caused certain inquiries to be made concerning them. Altogether the histories of 1,082 families, so selected, were obtained, and among other facts the number of previous children and the number of deaths which had occurred among them were ascertained. The total number of births in these famiies had been 4,574, and out of that number no fewer than 2,229 children had already died, practically all in infancy. Nearly half of the whole mass of children born in these families had died! Thus we seem to see that excessive infantile mortality goes not so much by districts as by families, and that it is the aggregation in certain districts of numbers of these "early-dying" families which gives to these places their high infantile death-rate. What it is that marks off one family from another in this way is a matter which requires careful investigation. The habit of improper feeding repeated with child after child, excessive dirt, drink, carelessness, may all be suggested; but it is clear that wherever one infant dies another is likely to die before long, so that those health missioners who so usefully devote their time to inculcating sanitary knowledge among the poor, may well take the occurrence of infantile deaths as an index, pointing out the families towards whose reformation their efforts may be most usefully directed.

### Soldiers' Deaths in Peace Time.

The admission regretfully made by General André, that the French Army loses a greater proportion of men by disease than the German, has drawn the attention of people to the health of the English Army. According to a writer in one of the newspapers, the health conditions of the British Army are even worse than those of the French. The actual number of deaths from disease in our army is more than twice that of the Germans, although the average peace strength of it is only a third. The figures for the three armies are given as follows:—

| | | | |
|---|---|---|---|
| Britain | 200,000 | 1,804 | 1·1 |
| France | 650,000 | 2,174 | 0·3 |
| Germany | 600,000 | 741 | 0·1 |

It might be claimed that the war had something to do with this excess of mortality in our army, but the statistics are taken for the ten years preceding the war. Thus the conditions are fairly equal, although British soldiers, of whom a greater proportion are on foreign service may, on account of the more frequent changes of climate which they have to undergo, be more susceptible to disease. But this being so, it is the more important that their clothing and the general conditions of their life should be carefully adapted to the place where they are serving. Complaints are made regarding the clothing worn at home, which is in many respects the same at Christmas as at midsummer, and complaints are also made of the barracks, many of which are old-fashioned, and far from being up to modern notions of sanitation. Whatever be the reasons of the greater mortality in our army, soldiers are national assets too valuable to be allowed to die of preventible disease, and it is to be hoped that public attention will be drawn to the subject.

